# Attention deficit–hyperactivity disorder is associated with allergic symptoms and low levels of hemoglobin and serotonin

**DOI:** 10.1038/s41598-018-28702-5

**Published:** 2018-07-06

**Authors:** Liang-Jen Wang, Ya-Hui Yu, Ming-Ling Fu, Wen-Ting Yeh, Jung-Lung Hsu, Yao-Hsu Yang, Wei J. Chen, Bor-Luen Chiang, Wen-Harn Pan

**Affiliations:** 1 Department of Child and Adolescent Psychiatry, Kaohsiung Chang Gung Memorial Hospital and Chang Gung University College of Medicine, Kaohsiung, Taiwan; 2 Institute of Epidemiology and Preventive Medicine, College of Public Health, National Taiwan University, Taipei, Taiwan; 3 Department of Food and Nutrition, Chung-Hwa University of Medical Technology, Tainan, Taiwan; 4 Institute of Biomedical Sciences, Academia Sinica, Taipei, Taiwan; 5 Graduate Institute of Humanities in Medicine, Taipei Medical University, Taipei, Taiwan; 6 Department of Pediatrics, National Taiwan University Hospital, College of Medicine, National Taiwan University, Taipei, Taiwan; 7 Department of Biochemical Science and Technology, College of Life Science, National Taiwan University, Taipei, Taiwan

## Abstract

This study investigated whether common comorbidities or biochemical factors, such as allergic disease, anemia, inflammation, and neurotransmitters, are singly or additively associated with an increased risk of attention deficit–hyperactivity disorder (ADHD). We recruited 216 children diagnosed with ADHD and 216 age-, sex-, height-, weight-, and class-matched controls from 31 elementary schools in Taipei, Taiwan. The International Study of Asthma and Allergies in Childhood questionnaire was used to measure allergic symptoms. Fasting venous blood was collected and analyzed for complete blood count, white blood cell differential count, immunoglobulin (Ig) E level, and serotonin (5-HT) level. The results showed that symptoms of both rhinitis (OR = 2.08, 95% CI = 1.42–3.05) and eczema (OR = 1.72, 95% CI = 1.02–2.88) were significantly associated with increased risk of ADHD. Children with ADHD showed considerably lower levels of hemoglobin (*p* = 0.001) and 5-HT (*p* < 0.001) and higher IgE level (*p* < 0.001) and eosinophil count (*p* = 0.001) than did control children. ADHD risk increased with the number of aforementioned biochemical risk factors present (one factor: OR = 1.87, 95% CI = 0.87–4.18; two factors: OR = 2.90, 95% CI = 1.29–6.48; three factors: OR = 4.47, 95% CI = 1.97–10.13; four factors: OR = 6.53, 95% CI = 2.43–17.57). Findings suggest that either ADHD’s etiology is multidimensional or the aforementioned conditions have shared etiology with ADHD.

## Introduction

Attention deficit–hyperactivity disorder (ADHD), one of the most frequent neuropsychiatric disorders found in children, is characterized by a lack of impulse control, inattention, and hyperactivity^1^. ADHD may have a substantial influence on children’s school performance, familial relationships, and social interactions^2,3^. Thorough investigation of ADHD’s complex etiology is vital for improving its diagnosis, prevention, and management^4^. ADHD is associated with various comorbidities, including allergic disease, immune dysregulation, neurotransmitter underproduction, anemia, and oxidative and endocrinological imbalances^5,6^.

Regarding the neurophysiological mechanisms, various allergic diseases, including allergic rhinitis, atopic dermatitis, and asthma, have already been associated with ADHD^7–11^. Immune responses resulting from these allergic diseases may affect the central nervous system (CNS) and predispose children with neurodevelopmental disorders, including ADHD^12^. Therefore, evaluating ADHD symptoms in children and adolescents with allergic diseases may facilitate ADHD identification^9^. Furthermore, identifying allergic diseases in children exhibiting ADHD symptoms provides an opportunity to control allergies. A recent randomized small-scale, placebo-controlled trial showed that rhinitis control combined with Ritalin administration can effectively alleviate ADHD endpoints^13^. Allergies result from immune responses that often involve chronic inflammation^14^. Immunoglobulin (Ig) E is an antibody found only in mammals, and an elevated IgE level is usually related to atopic diseases^15^. IgE-mediated allergic and inflammatory pathways are targets for intervention in the pathological processes of allergic diseases^16^. Furthermore, eosinophils, one of the five major leukocyte types, are responsible for tissue damage and inflammation in allergic diseases. However, little is known about whether the markers of allergy (i.e., peripheral IgE level or eosinophil count) may be linked to ADHD.

Atopic diseases, such as eczema, asthma, hay fever, and food allergies, are associated with anemia^17^. The most prevalent form of anemia is iron deficiency anemia. Low levels of serum ferritin, a biomarker of iron store, are correlated with risks of cognitive deficits and behavioral and psychiatric problems in pediatric populations^18^. A large-scale field study demonstrated that a lower ferritin level may be associated with parent-reported hyperactivity after controlling for confounding factors^19^. Furthermore, research, including a Taiwanese study, has indicated that iron deficiency or iron deficiency anemia may be a potential risk factor for neurodevelopmental disorders including ADHD^20,21^. Iron deficiency may also reduce the effectiveness of psychostimulant treatment in children with ADHD^22^. A routine complete blood count (CBC) can detect anemia, a late-stage marker of iron deficiency. This low-cost method may indicate poor iron nutrition and unsatisfactory response to psychostimulant treatment to prompt clinicians to treat these children with ADHD.

Serotonin (5-HT) is a monoamine neurotransmitter that induces metabolic effects in a variety of cell types both in the CNS and peripheral nervous system. A polymorphism on the 5-HT transporter gene (*5-HTTLPR*) can influence impulsivity in humans^23,24^. In addition, 5-HT can modulate several immunological events, such as activation, proliferation, cytokine secretion, and chemotaxis of leukocytes. The influence of 5-HT on immune cells may be associated with the pathologies of inflammatory diseases^25^ or atopic diseases (e.g., eczema or atopic dermatitis)^26,27^. Therefore, 5-HT deficits have been indicated as participating in the etiology of ADHD^28^. However, findings related to 5-HT circulation levels in ADHD patients have been inconsistent among previous case–control studies, with some studies indicating that children with ADHD tend to have lower blood levels of 5-HT^29–31^. However, other studies have not found this difference^32,33^. Therefore, additional studies with larger sample sizes (or greater statistical power) are required to confirm the association between 5-HT and ADHD.

Because ADHD etiology is multifaceted; each factor may make a partial contribution to ADHD susceptibility. Few studies have examined the joint effects of these factors. Understanding the complex relationships between ADHD and these conditions may elucidate ADHD pathogenesis and a comprehensive management plan. On the basis of the literature, we hypothesize that patients with ADHD have more allergic symptoms and higher levels of allergic and inflammation markers, such as IgE, histamine, CBC parameters, and 5-HT, compared with healthy controls. We aimed to investigate the dose–response relationship (cumulative effect) between the number of risk factors and susceptibility to ADHD. If links between the aforementioned risk factors and ADHD are established, routinely screening these underlying pathophysiological indicators may be helpful for devising a comprehensive treatment strategy for ADHD. Therefore, we conducted a case–control study by strictly matching age, sex, height, weight, and class to investigate whether ADHD is correlated with the aforementioned factors.

## Method

### Study participants

This study received approval from the Medical Research Ethical Committee of the Institute of Biomedical Sciences at Academia Sinica (AS-IBMS-MREC-92-01). All procedures involving participants were in accordance with the ethical standards of the institutional and national research committees and the Helsinki Declaration.

We mailed invitation letters to elementary schools in the Taipei metropolitan area and visited the schools agreeing to participate. Teachers distributed informed consent forms (ICFs). After completed ICFs were received from parents, we screened the students aged 8–10 years for potential ADHD on two validated scales: the Chinese version of the Conners Teacher Rating Scale (for teachers) and the Chinese version of the Werry–Weiss–Peters Activity Scale (for parents)^34^. Of all screened students, 287 students scored higher than the 85th-percentile values on both scales and were referred for further clinical evaluation. After being interviewed by a child psychiatrist on our research team, 252 students were diagnosed with ADHD according to the criteria of the Diagnostic and Statistical Manual, Fourth Edition, Text Revision (DSM-IV-TR). Control students were randomly selected from the same classes as the ADHD students were and matched by age, sex, height, and body weight (within a 5% range). After students with incomplete data were excluded, this analysis comprised 216 children with ADHD and 216 controls.

### Questionnaire, allergic symptoms, and biomarker assessment

We obtained information on family education levels, family income, lifestyle, and disease history of students and their parents through a questionnaire. Body weights and heights of the participating children were measured by trained technicians. We had parents fill out the International Study of Asthma and Allergies in Childhood (ISAAC) questionnaire^35^, and symptoms of asthma, rhinitis, and eczema were identified.

Approximately 10 mL of fasting venous blood was drawn from each child and stored in darkness throughout the biospecimen handling process. CBC analysis was performed at each school using EDTA-blood with a blood autoanalyzer (ADVIA 120; Bayer, Tarrytown, NY, USA; coefficients of variation [CVs]: WBC, 1.07%; RBC, 0.40%; HGB, 0.36%; HCT, 0.43%; MCV, 0.16%; MCH, 0.50%; MCHC, 0.52%; PLT, 1.72%). All serum and plasma samples were kept at −70 °C until analysis. We measured routine blood clinical chemistry and plasma C-reactive protein (CRP) concentrations by using the Olympus AU 600 Autoanalyzer (Olympus Corp., Tokyo, Japan; CV: 3.6–3.7%). IgE levels in the serum were analyzed with the radioimmunosorbent test and capsulated hydrophilic carrier (CAP) method (Pharmacia & Upjohn, Uppsala, Sweden; CV: 2.5–2.8%). Finally, we obtained histamine levels (CV: 2.2–9.2%) and 5-HT levels (CV: 3.6–17.9%) using ELISA kits (RE59121 for histamine and RE59221 for HT-5, IBL-America, Inc., USA).

### Statistical analysis

All statistical analyses were conducted using SAS (version 9.1; 2002, 2003, SAS Institute, Cary, NC, USA). We performed group comparisons between cases and controls using the chi-square test for categorical variables and *t* test for continuous variables. Logistic regression models were used to determine the relationships between each allergic disease (yes or no) and ADHD (present or absent). Eosinophil count and histamine, CRP, IgE, and 5-HT levels demonstrated a significant level of positive skewness. We applied arithmetic logarithmic transformations to approximate normal distributions for the aforementioned variables before preforming further statistical analyses. A *p* of < 0.05 was considered statistically significant. Bonferroni correction was applied to adjust for multiple testing in the group comparison, with regard to multiple biochemical indices (*p* = 0.05/16 = 0.003125).

Four indicators were identified: hemoglobin (Hb), eosinophil, IgE, and serotonin (5-HT). Risk conditions were defined by low levels of Hb and 5-HT, but high eosinophil count and IgE levels. Because no sex differences in these risk factors were noted among the children, we classified students into quartiles for each of the risk factors to compare effect sizes. We used logistic regression to determine the odds ratios of biochemical risk conditions as well as allergic conditions for their influences on ADHD. Potential confounders—namely jaundice after birth, mother with smoking habit, expenditure balanced with revenue, facing stressful situation (see Table 1)—were adjusted in each logistic regression model. Furthermore, we estimated the odds ratios for children with varying numbers of risk factors.

**Table 1:** Demographics of children in the ADHD group and in the control group.

Variables	Control (N = 216)	ADHD (N = 216)	*p-*value^a^
	Mean (SD)	Mean (SD)	
Age (years)	9.2 (1.8)	9.2 (1.7)	0.95
Height (cm)	135.2 (10.7)	135.2 (10.7)	0.93
Weight (kg)	33.6 (9.8)	33.6 (9.8)	0.95
Body mass index (kg/m^2^)	18.0 (3.1)	18.1 (3.1)	0.83
	N (%)	N (%)	
Male gender	186 (86)	186 (86)	1.00
**Grades**			
1–3 grades	121 (56)	121 (56)	1.00
4–6 grades	95 (44)	95 (44)	1.00
**Birth related conditions**			
Abortion symptoms in the 1st trimester	21 (10)	36 (17)	0.03
Maternal smoking habit in pregnancy	2 (1)	11 (5)	0.01
Jaundice after birth	14 (6)	35 (16)	0.001*
Presence of hypoxia at birth	6 (3)	16 (7)	0.03
Breast feeding	108 (50)	100 (46)	0.44
**Parental occupation**			
Non-blue collar	156 (72)	127 (59)	0.006
Blue collar	60 (28)	89 (41)	
**Having smoking habit currently**			
Parental	100 (46)	106 (49)	0.56
Maternal	11 (5)	30 (14)	0.002*
**Familial situation**			
Expenditure balanced with revenue	188 (87)	162 (75)	0.001*
Facing stressful situation	128 (59)	159 (74)	0.002*

^a^Chi-square test for groups comparisons of binary variables and t-test for groups comparisons of continuous variables; *Statistical significant after Bonferroni correction (p < 0.003125).

## Results

### Characteristics of study participants

Because children with ADHD and control children were matched by age, height, weight, body mass index, grade level, and sex, the distributions and means of these variables were comparable **(**Table 1**)**. Children with ADHD were more likely to have parents with lower education levels, blue-collar jobs, unstable incomes, higher levels of familial stress, conditions such as hypoxia or jaundice during or after delivery, and siblings with ADHD. Furthermore, children with ADHD tended to have mothers who were current smokers, smoked during pregnancy, and had experienced miscarriage symptoms in their first trimester of pregnancy.

### Relationship between ADHD and allergic diseases

**Figure 1:**
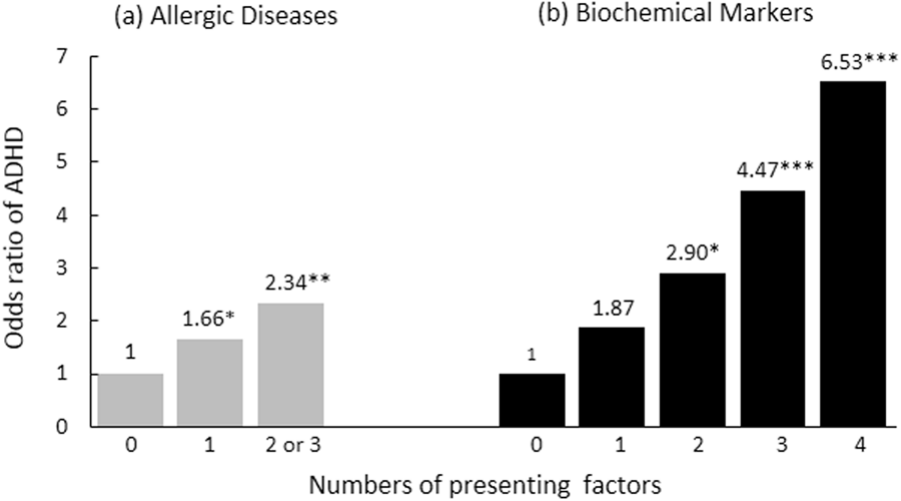
Effects of the number of allergic diseases **(a)** and clinical biochemical risk factors **(b)** on ADHD. (Numbers denoted above the bar are the exact odds ratios of corresponding groups; *p < 0.05, **p < 0.01, ***p < 0.001).

According to the ISAAC questionnaire, 24.5%, 61.6%, and 20.4% of our students with ADHD had symptoms of asthma, rhinitis, and eczema, respectively (Table 2). Rhinitis symptoms (OR = 2.08, 95% CI = 1.42–3.05) and eczema (OR = 1.72, 95% CI = 1.02–2.88) demonstrated a significant association with an increased risk of ADHD. Figure 1a shows that patients with at least one symptom of allergic diseases were more likely to receive an ADHD diagnosis (any one allergic disease: OR = 1.66, 95% CI = 1.06–2.60; two or three allergic diseases: OR = 2.34, 95% CI = 1.45–3.79).

**Table 2:** Associations between allergic diseases, biochemical markers and ADHD.

Allergic diseases	Control (N = 216)	ADHD (N = 216)	Logistic regression model	
	n (%)	n (%)	OR (95% CI)	*p-*value
Asthma	61 (28.2)	53 (24.5)	1.21 (0.79–1.86)	0.383
Rhinitis	94 (43.5)	133 (61.6)	2.08 (1.42–3.05)	<0.001
Eczema	28 (13.0)	44 (20.4)	1.72 (1.02–2.88)	0.040

Abbreviations: ADHD, attention-deficit hyperactivity disorder; OR, odds ratios; 95% CI, 95% confidence interval.

### Relationship between ADHD and biochemical indices

The biochemical profiles of children with ADHD and controls are listed in Table 3. Significantly lower Hb and 5-HT levels were observed in students with ADHD. By contrast, these children had significantly higher IgE levels and eosinophil counts than the control children. We did not observe significant differences in histamine or CRP levels or CBC parameters, except Hb levels.

**Table 3:** Comparisons of biochemistry profile between ADHD patients and healthy control children.

Biochemical indexes	Control (N = 216)	ADHD (N = 216)	*Statistic* ^b^	*p-*value
	Mean (SD)	Mean (SD)		
RBC (10^12^/L)	4.70 (0.27)	4.64 (0.32)	2.259	0.024
Hb (g/dL)	13.30 (0.85)	13.00 (0.80)	3.466	0.001*
MCV (fL)	84.10 (3.6)	83.00 (4.7)	2.730	0.007
MCH (pg/cell)	28.20 (1.5)	28.10 (1.9)	0.392	0.70
MCHC (g/dL)	33.60 (1.4)	33.90 (1.4)	1.619	0.11
WBC (10^9^/L)	7.02 (1.71)	7.38 (2.11)	1.919	0.06
Neutrophil (%)	49.20 (10.5)	49.80 (9.8)	0.682	0.50
Lymphocyte (%)	39.10 (9.5)	38.30 (8.9)	0.902	0.37
Monocyte (%)	6.83 (1.9)	6.77 (1.83)	0.346	0.73
Eosinophil (%)^a^	3.78 (2.67)	4.93 (3.62)	3.364	0.001*
Basophil (%)	0.61 (0.85)	0.57 (0.40)	0.553	0.58
Platelet (10^9^/L)	310.00 (66)	314.00 (66)	0.585	0.56
Histamine (nmol/L)^a^	9.54 (9.18)	9.99 (9.27)	0.461	0.65
CRP (mg/L)^a^	0.96 (1.66)	1.35 (2.71)	1.470	0.14
IgE (KU/L)^a^	262.38 (420.27)	367.44 (483.36)	3.559	<0.001*
Serotonin (ng/mL)^a^	191.61 (88.26)	164.25 (88.39)	4.025	<0.001*

^a^Log transformation has been done for statistical testing; ^b^Chi-square test for groups comparisons of binary variables or t-test for continuous variables, *Statistical significant after Bonferroni correction (p < 0.003125).

We defined the cutoff points for biochemical risk factors using quartile cutoff points. Although these points were situated in the subclinical (normal) range, logistic regression models indicated that Hb < 12.6 g/dL, 5-HT < 113.57 ng/mL, IgE > 367 KU/L, and eosinophil >5.85% were all significantly associated with an increased ADHD risk (Table 4). Furthermore, children with one or more of the aforementioned biochemical risk factors were more likely to be diagnosed with ADHD (any one factor: OR = 1.87, 95% CI = 0.87–4.18; any two factors: OR = 2.90, 95% CI = 1.29–6.48; any three factors: OR = 4.47, 95% CI = 1.97–10.13; four factors: OR = 6.53, 95% CI = 2.43–17.57; Fig. 1b).

**Table 4:** Associations between biochemical markers and ADHD.

Biochemical marker at risk	Control (N = 216)	ADHD (N = 216)	Logistic regression model	
	n (%)	n (%)	OR (95% CI)	*p-*value
Hb (<12.6 g/dL)	49 (22.7)	69 (31.9)	1.60 (1.04–2.45)	0.031
Eosinophil (>5.85%)	41 (19)	67 (31)	1.92 (1.23–3.00)	0.004
IgE (>367 KU/L)	44 (20.4)	62 (28.7)	1.57 (1.01–2.45)	0.045
Serotonin (<113.57 ng/mL)	45 (20.8)	63 (29.2)	1.57 (1.01–2.43)	0.046

Abbreviations: ADHD, attention-deficit hyperactivity disorder; OR, odds ratios; 95% CI, 95% confidence interval.

## Discussion

To our knowledge, this study is the first to examine the relationship between ADHD and allergies along with multiple biochemical indices related to immune-mediated inflammation. Compared with control participants, children with ADHD had higher prevalence of allergic diseases, higher eosinophil counts, higher IgE levels, lower blood 5-HT concentration, and lower Hb levels. The higher the number more risk factors was, the higher the odds of being diagnosed with ADHD were. This finding suggests the coexistence of ADHD and several clinical conditions, including allergic diseases; biomarkers of immunological responses and the associated conditions, such as anemia; and lower 5-HT levels.

The results of our study indicate a significantly higher prevalence of rhinitis and eczema in the ADHD group than in the control group. This finding is consistent with that of a previous meta-analysis on allergic diseases and ADHD, although only eczema was independently related to ADHD in that study^36^. Furthermore, our study showed the number of allergic disease symptoms had a dose–response effect on ADHD, suggesting that patients with ADHD had more allergic conditions than their counterparts without ADHD. Various reasonable biological mechanisms linking ADHD and allergies were proposed^37^. Allergic reactions result in an increase in inflammatory cytokines from TH2 or TH17 cells^38^, which may stimulate the neuroimmune mechanism in the brain circuits, such as the prefrontal cortex and the anterior cingulate cortex, associated with emotional and behavior control^39,40^. ADHD symptoms resemble cognitive disturbances caused by functional and structural changes in the aforementioned two brain circuits. Although neither the existing literature nor this study could provide a chronological sequence from the development of atopic disease to that of ADHD, we found total IgE levels and percentage of eosinophils to be elevated in children suffering from ADHD. Because it is unlikely that ADHD causes allergy, these findings support the hypothesis that allergies precede or coexist with ADHD because of some shared etiology. Although it is not confirmed at the present time whether or how allergy is involved in the etiology of ADHD, allergic symptom assessment may be considered in the comprehensive appraisal and management of ADHD in children.

Iron deficiency may also result in an insufficient supply of neurotransmitters because iron is a cofactor of tyrosine hydroxylase and tryptophan hydroxylase^41^. Previous studies have also found iron deficiency to alter dopamine transporter function in rat striatum^42^. Not only is iron involved in neurotransmitter production but iron deficiency may also contribute to the development of ADHD by disrupting the oxygen supply to the brain^43^ and disturbing growth and cognitive ability^44^. Studies related to the iron status of patients with ADHD and controls have been controversial^22,45,46^. We did not directly assess ferritin levels, but rather performed a routine CBC assessment. We observed significantly lower Hb levels in children with ADHD. Although the threshold points for Hb (12.6 g/dL at 25th-percentile) did not reach the criterion for anemia, this finding indicates that subclinical anemia may be associated with certain aspects of ADHD. Iron deficiency is the only possible cause of microcytic anemia. The lower Hb levels in children with ADHD may have also resulted from other nutrient deficiencies, such as folate, vitamin B6 or B12, or other inflammatory or parasitic conditions. Moreover, asthma and infectious diseases have also been associated with anemia^17,47,48^, implying that allergies (immune responses) and anemia may share a biological process and environmental factors. Determining causes of anemia is not difficult clinically and may help to improve the overall well-being of patients with ADHD.

The CNS inhibitory neurotransmitter 5-HT correlates with impulse control and emotions^23,49^. We found that children with ADHD had lower 5-HT levels compared with healthy controls. This finding agrees with those of previous studies^29–31^ but disagrees with those of others^32,33^. Relative to previous studies that investigated a similar topic, the current study featured a larger sample and matched controls more strictly. Therefore, we believe that our study possessed greater statistical power compared with previous small-scale studies. Furthermore, a recent study indicated that the peripheral function of the 5-HT metabolic pathway can serve as a significant predictor of aggressive behavior^50^. It has also been found that 5-HT may also exert effects on immune cells and link to the pathologies of inflammatory diseases^25^ or atopic diseases (eczema or atopic dermatitis)^26,27^. Therefore, certain characteristics of children with ADHD, such as impulsivity, temper tantrums, or aggression, may be partially related to lower peripheral 5-HT levels.

Here, although each single factor showed significant differences between the ADHD group and control group (*p* < 0.05), the discriminative validity of each was not robust. We found that children with more of the aforementioned biochemical risk factors were more likely to be diagnosed with ADHD. The dose–response relationship between the number of risk factors and susceptibility to ADHD supports either the idea that the etiology of ADHD may be multifaceted or that ADHD coexists with allergy, immunological stress, anemia, and low 5-HT levels because of a shared etiology^41,51^. Notably, several of these biomarkers may also interact with each other. Studies^25,52^ have demonstrated that several nutritional factors and allergic reactions are associated with serotonin levels. Additional studies are required to clarify the underlying mechanisms of the relationships between these biochemical abnormalities and ADHD and assess their predictive values and applicability in clinical settings. Because allergic diseases and low levels of Hb and 5-HT may be modifiable through dietary intervention, nutritional supplements, or medical treatment, clinicians should monitor these factors in children exhibiting ADHD symptoms. Adequate correction for these risk factors may be beneficial to some children diagnosed with ADHD or who respond poorly to ADHD drug therapy (maybe phenocopies of ADHD).

This study has several limitations. First, this was a case–control study, and the causal relationships among ADHD, allergic symptoms, and 5-HT and Hb levels warrant further investigation. Second, although ADHD diagnoses were confirmed using the DSM-IV-TR, the ADHD severity and neuropsychological functions of the participants were not assessed. The complex relationships among clinical characteristics (e.g., severity of ADHD symptoms, comorbidity, or neurocognitive deficits) and severity of allergic diseases and biomarkers consequently remain uncertain. Third, allergy symptoms were only defined using the ISAAC. Nonetheless, findings on IgE and eosinophil were consistent with the questionnaire results. In addition, this study only measured Hb concentration in the blood. We cannot confirm whether it is anemia or iron deficiency that is involved in ADHD etiology. Finally, not only 5-HT but also other neurotransmitters (e.g., dopamine or norepinephrine) are involved in the pathophysiology of ADHD^38^; however, we did not determine the levels of neurotransmitters other than 5-HT.

In conclusion, ADHD is associated with several comorbidities, including allergic symptoms, subclinical anemia, and low 5-HT levels; furthermore, the number of biochemical risk factors is associated with the presence of ADHD in a dose–response manner. Thus, either ADHD etiology is potentially multidimensional or ADHD has shared etiology with the other included clinical conditions. Future longitudinal studies should investigate the causal relationships between these subclinical indicators and ADHD and determine whether ADHD risk can be modified by appropriately managing allergic symptoms and Hb and neurotransmitter levels.
